# White Matter Deterioration May Foreshadow Impairment of Emotional Valence Determination in Early-Stage Dementia of the Alzheimer Type

**DOI:** 10.3389/fnagi.2017.00037

**Published:** 2017-03-01

**Authors:** Ravi Rajmohan, Ronald C. Anderson, Dan Fang, Austin G. Meyer, Pavis Laengvejkal, Parunyou Julayanont, Greg Hannabas, Kitten Linton, John Culberson, Hafiz M. R. Khan, John De Toledo, P. Hemachandra Reddy, Michael O’Boyle

**Affiliations:** ^1^Department of Pharmacology and Neuroscience, Texas Tech University Health Sciences CenterLubbock, TX, USA; ^2^Department of Electrical and Computer Engineering, Texas Tech UniversityLubbock, TX, USA; ^3^Department of Human Development and Family Studies, Texas Tech UniversityLubbock, TX, USA; ^4^School of Medicine, Texas Tech University Health Sciences CenterLubbock, TX, USA; ^5^Department of Neurology, Texas Tech University Health Sciences CenterLubbock, TX, USA; ^6^Department of Public Health, Texas Tech University Health Sciences CenterLubbock, TX, USA; ^7^Department of Family Medicine, Texas Tech University Health Sciences CenterLubbock, TX, USA; ^8^Garrison Institute on Aging, Texas Tech University Health Sciences CenterLubbock, TX, USA; ^9^Cell Biology and Biochemistry, Texas Tech University Health Sciences CenterLubbock, TX, USA; ^10^Speech, Language and Hearing Sciences, Texas Tech University Health Sciences CenterLubbock, TX, USA

**Keywords:** Alzheimer, chimeric faces, brain networks, DTI, fMRI, emotional valence

## Abstract

In Alzheimer Disease (AD), non-verbal skills often remain intact for far longer than verbally mediated processes. Four (1 female, 3 males) participants with early-stage Clinically Diagnosed Dementia of the Alzheimer Type (CDDAT) and eight neurotypicals (NTs; 4 females, 4 males) completed the emotional valence determination test (EVDT) while undergoing BOLD functional magnetic resonance imaging (fMRI). We expected CDDAT participants to perform just as well as NTs on the EVDT, and to display increased activity within the bilateral amygdala and right anterior cingulate cortex (r-ACC). We hypothesized that such activity would reflect an increased reliance on these structures to compensate for on-going neuronal loss in frontoparietal regions due to the disease. We used diffusion tensor imaging (DTI) to determine if white matter (WM) damage had occurred in frontoparietal regions as well. CDDAT participants had similar behavioral performance and no differences were observed in brain activity or connectivity patterns within the amygdalae or r-ACC. Decreased fractional anisotropy (FA) values were noted, however, for the bilateral superior longitudinal fasciculi and posterior cingulate cortex (PCC). We interpret these findings to suggest that emotional valence determination and non-verbal skill sets are largely intact at this stage of the disease, but signs foreshadowing future decline were revealed by possible WM deterioration. Understanding how non-verbal skill sets are altered, while remaining largely intact, offers new insights into how non-verbal communication may be more successfully implemented in the care of AD patients and highlights the potential role of DTI as a presymptomatic biomarker.

## Introduction

Alzheimer disease (AD) is marked by a progressive decline in cognitive functions. As this occurs, the affected individual becomes less capable of understanding the world around them. This occurs in part due to a loss of ability to interpret the body language and facial expressions of others. Face-processing represents a group of complex cognitive operations in which information is extracted from facial features in such a way that the observer is able to perceive pertinent information about the person they are viewing. Previous studies of moderate-stage AD patients showed that their ability to interpret basic emotional cues from faces is largely intact (Roudier et al., 1998; Luzzi et al., 2007). However, dysfunctions are detectable (Albert et al., 1991; Cadieux and Greve, 1997; Hargrave et al., 2002). Luzzi et al. (2007) showed that the ability to interpret emotional cues directly correlated to participants’ performance on constructional praxis and visuospatial memory tasks, which are examples of non-verbally-mediated skills, in early-moderate-stage AD.

These findings, combined with the observation that stroke participants’ performance on certain visuospatial tests correlates with the degree of localization of damage to the right parietal lobe (Luzzi et al., 1998), suggest that activity of the right parietal lobe is directly related to the interpretation of emotional cues. This concurs with both the current clinical and pathological picture of AD, which shows that non-verbal memory loss, as well as damage to the right parietal lobe, is a late-stage finding (Braak et al., 2006; Ally et al., 2009).

We attempted to correlate the non-verbal skills of participants with early-stage Clinically Diagnosed Dementia of the Alzheimer Type (CDDAT) with findings from both functional magnetic resonance imaging (fMRI) and diffusion tensor imaging (DTI) measures during a non-verbally mediated face-processing task. Participants underwent a well-established, standardized, non-verbal skill test (the Rey-Osterrieth Complex figure B, visuospatial memory test (ROCFB-VMT); Luzzi et al., 2007) before entering the scanner to establish their visuospatial memory baseline. Participants then performed a variation of the Chimeric Face Test (CFT; Levy et al., 1983) that we created called the “emotional valence determination task (EVDT)” while in the scanner.

CDDAT patients were expected to perform as well as or slightly worse than neurotypicals (NTs) on the EVDT. Neuroimaging data was expected to show functional under-activation within the frontal (inferior frontal; left middle frontal) and parietal lobes (bilateral supramarginal gyri and left precuneus), given their association with non-verbal emotional processing, as well as increased activation of the bilateral amygdala and right anterior cingulate cortex (r-ACC), reflecting a greater reliance on these structures, during the EVDT (Nakamura et al., 1999; Fusar-Poli et al., 2009). Likewise, we hypothesized that DTI may show some deterioration of white matter (WM) tracts within frontoparietal connections, but not within amygdaloidal or cingulate tracts. Performance on the EVDT was expected to correlate to ROCFB-VMT performance. We hypothesized that this represents a relationship between emotional valence determination and visuospatial memory. By investigating the potential association of early-stage AD neuroimaging findings and non-verbal skill performance, we assessed the usefulness of these tests as cost effective supportive diagnostic markers of AD.

As may be expected, most task-related studies of AD focused on memory and displayed a pattern of early compensation followed by decreased activity. Pariente et al. (2005) noted increased activation during episodic memory encoding and recall in the posterior cingulate cortex (PCC), precuneus, parietal lobe and frontal lobe in early-stage AD patients with a mean Mini Mental State Exam (MMSE) score of 25 ± 1.8. Grady et al. (2003), Dickerson et al. (2005), Celone et al. (2006) and Zhou et al. (2008), on the other hand, observed decreased or even absent activation relative to NTs in the same regions in early-stage AD patients with mean MMSE scores of 21.1 ± 3.1, 22 ± 5, 21.3 ± 2.7, respectively. These findings support the assertion of Risacher and Saykin (2013) that increased activation occurs early on in an attempt to compensate, but ultimately leads to decreased activation once the disease burden is too great. We reasoned that this pattern of initially increased activation leading to decreased activation could be demonstrated for other cognitive domains as well because it represents a fundamental mechanism of the energetics of neurodegeneration. For a comprehensive review of AD discoveries through neuroimaging see Risacher and Saykin (2013).

Of equal importance is the understanding of how WM tracts are affected by the disease. WM alterations appear to parallel gray matter (GM) changes in that cortical abnormalities are greater in posterior brain regions relative to anterior regions at the early-stages of AD (Arnold et al., 1991; Braak and Braak, 1995). When the disease progresses, the neurofibrillary pathology advances from limbic to frontal structures, into higher-order association cortices, and finally into primary sensorimotor areas, which correlates with the clinical manifestations of AD (Braak and Braak, 1995). DTI-based tractography studies (Fellgiebel et al., 2005) and whole-brain DTI studies (Medina et al., 2006; Rose et al., 2006; Zhang et al., 2007) have consistently shown that fibers located deep in the posterior WM (e.g., the superior longitudinal fasciculus (SLF) and the posterior cingulum bundle (PCB)) are affected in patients with AD and mild cognitive impairment (MCI), a common precursor to AD. Bartzokis et al. (2003, 2004) have proposed that this may occur because as brain development takes place, later myelinated regions (cortical association areas) have fewer oligodendrocytes supporting greater numbers of axons (Bartzokis, 2004). DTI findings of decreased WM integrity in later myelinated regions at the onset of AD support this “reversed demyelination” construct (Medina and Gaviria, 2008). Furthermore, Huang et al. (2007) delineated a neuroanatomical pattern of functional alterations showing that changes in WM diffusion in parietal lobes correlated with scores of visuospatial skills.

Research on amnestic type MCI (aMCI) populations using neuropsychological tests of declarative memory extend this trend as they have demonstrated significant correlations between declining performance and decreases in posterior WM fractional anisotropy (FA), particularly in the PCB (Fellgiebel et al., 2005, 2008, Rose et al., 2006). Recalling the proposal of Bartzokis et al. (2004), disruptions in transcortical connectivity may serve as early contributors to the pathophysiology of dementia, as the observed WM deteriorations were embedded beneath cortical GM that is often affected early within the disease course. For a more in-depth review of DTI findings in AD, see Medina and Gaviria (2008).

Although memory deficits are a hallmark characteristic of AD, there may be little to gain from testing face-processing related to familiarity that isn’t already known (Sperling et al., 2003; Golby et al., 2005; Winchester, 2009; Donix et al., 2013). On the other hand, the fundamentals of face-processing (e.g., emotional valence) have received little attention (Job, 2012) and warrant further investigation. The use of neuropsychological tests in combination with neuroimaging techniques stresses the importance of attempting to integrate pathological observations with clinical symptoms. In doing so, we are able to reinforce findings from either end of the spectrum to more efficiently develop our understanding of the disease. In order to investigate a cognitive operation as complex as face-processing, it will be necessary to use appropriate tests that can isolate its specific subdivisions. This is particularly important when performing an fMRI investigation, as some studies suggest that face-processing and recognition occurs in fractions of a second (Vuilleumier and Schwartz, 2001; Batty and Taylor, 2003). If such factors are not properly accounted for, it would be very difficult to remove these confounds from the fMRI data as the canonical hemodynamic response curve peaks between 4–6 s after the presented stimuli, making it far too slow to tease out these processes (Poldrack et al., 2011).

To this end, the EVDT was of particular value since it is a variation of the CFT. The CFT was originally developed by Levy et al. (1983) “to index functional cerebral asymmetry for processing facial characteristics.” It was shown to be highly reliable in detecting differences between right- and left-handers as well as being stable with regard to individual differences in perceptual asymmetries (Levy et al., 1983).

CFTs have previously been used to investigate asymmetries in the processing of emotions from facial expressions. Early works by Albert et al. (1991) and Cadieux and Greve (1997) suggested that by the moderate-stage, AD patients were impaired in recognizing emotions, but the authors noted that they could not rule out confounds “due to the deficits in recognizing non-emotional facial features and in verbal processing”. Roudier et al. (1998) demonstrated that moderate-stage AD patients could accurately recognize when different emotions were displayed using the same human face. Hargrave et al. (2002) reconciled this difference through the use of a “same-different” emotion differentiation task in which participants were presented with a pair of photographs of different people and were asked to state if the two photographs in the pair were depicting the same or different emotions. From this, it was determined that moderate-stage AD patients do, in fact, have difficulty differentiating emotions independent of verbally-mediated and non-emotional facial features. Indersmitten and Gur (2003) then determined that two separate circuits likely underlie emotional processing in facial asymmetries and, while the left hemiface/right cerebral hemisphere circuit was dominant for expressions of sad, fearful and happy, the right hemiface/left cerebral hemisphere proved to be more efficient on task performance in NTs.

Then, Luzzi et al. (2007) used a variation of the CFT called the Mona Lisa test (MLT) wherein they determined that when using a cartoon face, emotional valence determination was impaired in some participants with moderate-stage AD, but not significantly across the entire cohort. Luzzi et al. (2007) found that in those with impaired recognition of facial emotions, the impairment correlated to poor performance on constructional praxis and non-verbal memory test, but not to the verbal fluency test (VFT) or MMSE score. Therefore, the ability to recognize facial emotions correlated to non-verbal performance. Finally, a fMRI meta-analysis by Fusar-Poli et al. (2009) discovered that the processing of facial expressions for emotional valence was associated with neural activation in the parietal and frontal cortices in NTs, thereby making these ideal regions of interest for investigation of the emotional subdivision of face-processing.

All things considered, we chose to use a “same-different” task similar to Hargrave et al. (2002) to observe changes in brain activity and connectivity in early-stage CDDAT patients as it represents the most clinically relevant task given its translatability to real life scenarios. Their work did not incorporate neuroimaging techniques, however, representing a gap in knowledge which we intended to fill with the current study. The works of Roudier et al. (1998), Indersmitten and Gur (2003), Luzzi et al. (2007) and Fusar-Poli et al. (2009) established crucial observations that were necessary to proceed in such a manner as without them there would be too many potential confounds to consider using depictions of actual human faces, let alone a series of different faces, to assess emotional processing through neuroimaging. Additionally, by using non-chimeric variations of faces based on the Mattingley et al. (1993) chimeric faces, we more directly assessed the emotional valence components of face-processing than we could through the original CFT.

The ROCFB served as an ideal cognitive test for investigating the non-verbal underpinnings of emotional valence determination because it had been described by Luzzi et al. (2011) to be “a valid instrument to assess non-verbal memory in adults and in the elderly”. Building upon the findings of Luzzi et al. (2007), we investigated the relationship between the ROCFB-VMT and the EVDT. We assessed the potential relationship between visuospatial memory (as measured by the ROCFB-VMT) and the EVDT to determine if the ability to discern emotional states from facial expressions is rooted in the brain’s ability to recognize and retain visuospatial relations between shapes.

## Materials and Methods

### Participant Identification and Selection

The University Medical Center Departments of Neurology, Family Medicine, and Geriatrics saw 171 patients for complaints of “memory problems” or “dementia” spanning a 6-month period (November 2015–April 2016). Potential participants were identified and classified into their respective categories by physician assessment. Of that, 100 were determined to have AD or its precursor, aMCI, in accordance with the guidelines outlined by McKhann et al. (2011) for “dementia due to AD” or “MCI due to AD” (Albert et al., 2011). Those in the possible early-stage AD category were selected based on an MMSE score (Folstein et al., 1975) of 26–21 in accordance with National Institute for health Care and Excellence guidelines (National Institute for Health and Care Excellence, 2011). Twenty of these patients were determined to fit our inclusion/exclusion criteria, four of whom agreed to participate. All CDDAT participants had an existing medical MRI scan interpreted by a radiologist within the last 5 years prior to the study. These individuals were grouped into the CDDAT group. Detailed inclusion/exclusion criteria for the CDDAT group are listed below:

Inclusion criteria

- The diagnosis of AD was made in line with international diagnostic criteria (Albert et al., 2011; McKhann et al., 2011) on the basis of clinical history, neurologic, psychiatric, neuropsychological evaluation and hematologic screening for dementia (including liver and kidney function, thyroid function, folic acid and B12 vitamin levels, neoplastic markers), electroencephalogram, carotid ultrasonography, and neuroimaging studies (computed tomography and/or magnetic resonance) as determined by the attending physician.
- Mean general cognitive decline was evaluated with the MMSE (Folstein et al., 1975).
- All participants gave their informed consent to participate in the study.

Exclusion criteria

- Inability to understand the instructions from the principal investigator (PI) or research conductors.
- History of substance abuse.
- History of neurological deficits not related to AD.
- Inability or refusal to give informed consent.
- Motor, visual, or hearing impairment that precluded ability to perform cognitive testing assessments.
- Failure to pass the MRI safety checklist guidelines.

Ten age-matched cognitively normal participants (5 males, 5 females) were recruited from a nearby senior living center and were grouped into the NT group. NTs were required to have an MMSE score >27 and meet all inclusion and exclusion criteria stated above, except for that concerning the existence of Alzheimer-related pathology. One male participant was retroactively removed after receiving a diagnosis of normal pressure hydrocephalus. One female participant from the NT group was removed due to a scanner script runtime error that prevented the EVDT from being run. Therefore, there were 4 CDDAT (1 female, 3 males) and 8 NT (4 females, 4 males) participants in this study. All participants were right-handed, except for 1 non-right-hand-dominant NT female, as confirmed by the Edinburgh Handedness Inventory-revised (EHI-r; Williams, 1986). The non-right-hand-dominant NT female that was kept in the study was kept because the participant was shown to have no significant difference in either their accuracy, reaction time (RT), or within-group contrast mapping for either fMRI or DTI measures when compared to the rest of the control group.

Participants were informed that their participation was voluntary and they may withdraw from the study at any time and that their refusal to participate would have no impact on their level of care. This study was carried out in accordance with the recommendations of Texas Tech University Human Protections Internal Review Board with written informed consent from all subjects. All subjects gave written informed consent in accordance with the Declaration of Helsinki. The protocol study was approved by the Texas Tech University Human Protections Internal Review Board.

#### Sample Size

A 2004 meta-analysis by Henry et al. (2004) demonstrated an effect size of *r* = 0.73 for a test of semantic fluency in patients with Dementia of the Alzheimer Type (DAT). From this it was determined that an assumed effect size of *r* = 0.8 would achieve 80% power with a minimum sample size of 4. Our behavioral analyses, therefore, seek to determine if an impairment on either the EVDT or ROCFB-VMT is greater than or equal to the known deficit of verbal fluency, as this currently represents the most consistent and pronounced clinical manifestation of the disease at this stage. Effect size for each test was calculated using Cohen’s *d* and converted to its r-equivalent (Cohen, 1992).

The number of necessary participants was based on the Desmond and Glover (2002) report that single voxel-level power of 80%, corrected for multiple comparisons can be achieved with a sample size of 24 per group. According to Desmond and Glover (2002), “Sample size increases power because the standard error of the mean decreases by the square root of *N*” (*N* = the number of subjects). In our study, we had a minimum of four subjects within each group. Therefore, we may expect to reach of 32.7% power at the single voxel level (i.e., since we have 1/6 of the number of subjects we will reach 41% of the power achieved by having 24 per group or 33% overall; the square root of 4/24 = 0.41*0.8 = 0.328). As such, even by the most conservative of estimates (assuming a power of 10 exponential) our minimum detection for 80% power is 316 voxels (80%/32%= 2.5; 10^2.5^= 316). Given that this is a region-of-interest-based investigation (ROI) >1000 voxels, however, we may reasonably expect to surpass 80% power for each ROI with a sample size of ≥4 per group.

#### Participant Demographics

Participant demographics are summarized in Table 1. We recruited four right-handed Caucasian patients (3 males, 1 female) with a diagnosis of aMCI or early-stage AD between the ages of 73–93 (median age 83.5 ± 8.4) with MMSE scores between 26–23 (median of 24.5 ± 1.3). Two of the males (MMSE scores of 26 and 25) had an existing diagnosis of “MCI due to AD” (Albert et al., 2011). The remaining male and female had an existing diagnosis of “dementia due to AD” (MMSE scores of 24 and 23, respectively; McKhann et al., 2011). Eight (7 right-handed and 1 non-right-hand-dominant) cognitively normal Caucasian participants (4 males, 4 females; all with MMSE scores of 30) between the ages of 79–91 (median age 80 ± 4.0) also participated. Handedness was determined using the EHI-r (Williams, 1986).

**Table 1:** **Participant demographics**.

Group	Age (median ± stdev)	Sex	Handedness	Race	MMSE (median ± stdev)
CDDAT (*n* = 4)	83 ± 8	1 F, 3 M	4 R	4 CA	24.5 ± 1.3
NT (*n* = 8)	80 ± 4	4 F, 4 M	7 R, 1 nR	8 CA	30 ± 0

*CDDAT, clinically diagnosed dementia of the Alzheimer type; NT, neurotypical; F, female; M, male; R, right-handed; nR, non-right-handed; CA, Caucasian*.

There was no significant difference in median age (*p* = 0.561), but there was an observable difference in median MMSE score for the two groups (*p* = 0.014). While there may be some concern given our lack of equal sex-distribution (1 female and 3 males) within the CDDAT group, no differences in performance were discernable based on sex within our NT group (4 females, 4 males) across either cognitive test (ROCFB-VMT or EVDT) for either accuracy or RT (data not shown) nor for within-group sex-based contrast mapping for fMRI or DTI (data not shown).

### Administration of Rey Osterrieth Complex Figure B Visuospatial Memory Test

Participants were first asked to copy the ROCFB as accurately as possible, without tracing it, on a different sheet of paper that had the same dimensions as the paper containing the figure. The size of the model was 5.5 cm × 8 cm, and the sheet of paper on which it was printed was half the size of an 8.5′′ × 11′′ sheet. Participants were then asked to reproduce the ROCFB from memory on a different sheet of paper after an interval of 5 min, during which they were engaged with the VFT (Luzzi et al., 2007). The rate of retention was calculated as a percentage of the score obtained on reproduction from memory compared to the original figure-copying score. Digital photographs were taken of the drawings produced by participants to ensure accuracy of scoring. The test was administered and scored following the protocol detailed in Luzzi et al. (2011).

### Imaging Methodology and Analyses

#### Stimulus Presentation and Participant Response

**Figure 1:**
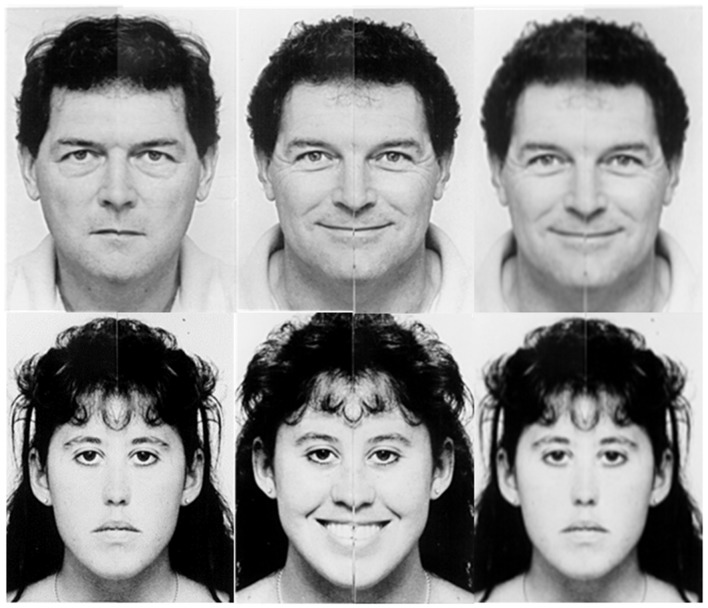
**Examples of the emotional valence determination test (EVDT).** EVDT examples in response to the question: “Are the emotions the same?” (left) “yes”; two neutral faces. (center) “yes”; two happy faces. (right) “no”; happy (top) and neutral (bottom).

After completing the ROCFB-VMT, all participants were briefly trained on the EVDT before entering the scanner to ensure task comprehension by allowing them to practice on a single example trial of each condition type. Pairs of faces based on the chimeric faces of Mattingley et al. (1993) were presented in an event-related design for the EVDT using Eprime 2.0. Presentation order was counterbalanced across participants using a Latin square design. The question “Are the emotions the same?” was displayed upon the screen for 4 s inside the scanner. A set of 40 trails were randomized and displayed for 4 s (s) each with a jittered inter-stimulus interval (ISI) randomized for five time points between 800–1200 ms, by 100 ms apiece. Participants responded using a fiber optic controller held in the right hand where button 1 was pressed by the index finger and button 2 was pressed by the middle finger. Participants pressed one of two buttons to indicate Yes (button 1) or No (button 2) in response to the question. An example of this test is given in Figure 1.

#### Scanning Parameters

All images were acquired with a 3T Siemens MR system (Skyra, Germany) at the Texas Tech Neuroimaging Institute. The T1 anatomic scan parameters were: TR: 1900 ms; TE: 2.49 ms; FOV: 240; Flip angle: 9, Voxel size = 0.9 × 0.9 × 0.9 mm; slice number: 192. The fMRI parameters were: TR: 2500 ms; TE: 20.0 ms; FOV: 231; Flip angle: 75, Voxel size = 2.5 × 2.5 × 3.0 mm; slice number: 41. In each fMRI dataset, there are 80 volumes. The DTI parameters were: TR: 5000 ms; TE: 95 ms; FOV: 220; B/W: 1562, Voxel size = 1.7 × 1.7 × 4.0 mm; slice number: 32. There were 64 directions for DTI.

#### Image Preprocessing

##### fMRI

Image preprocessing steps included removing non-brain structures by Brain Extraction Tool (BET), motion correction by using Motion Correction for FSL Linear Registration Tool (MCFLIRT), temporal high-pass filtering with a cutoff period of 24 s, spatial smoothing with a 5 mm Gaussian full width, half maximum (FWHM) algorithm, and co-registering of the functional images to the high resolution T1 structure images in their native space using boundary border registration (BBR) and FSL Linear Registration Tool (FLIRT; Jenkinson and Smith, 2001; Jenkinson et al., 2002) at 12 degrees of freedom to the Korean Normal Elderly (KNE96; Lee et al., 2016) standard brain space.

##### DTI

FSL Diffusion Toolbox 3.0 (FDT) from FMRIB Software Library (FSL 5.0.5) was used to complete the construction of and preprocessing for all anatomical brain networks for all subjects. It processed DICOM/NIfTI files into diffusion metrics (e.g., FA) that were ready for statistical analysis at the voxel-level after performing corrections for image alignment and artifact clean-up (Top-Up) and local field distortions (eddy current correction; Smith et al., 2004).

#### Image Processing

##### fMRI

fMRI data processing was carried out using FMRI Expert Analysis Tool (FEAT) Version 6.00, part of FSL^1^ (Worsley, 2001). The time series for the behavioral events of the EVDT were analyzed for the following conditions:

For “presentation of stimuli scenarios” (i.e., when the participant was shown two happy faces, two neutral faces, or one of each)—brain activity from the first 2 s following the presentation of a stimulus was recorded for each trial. In the event a participant responded within <2 s, the interval between the presentation of the stimulus and 250 ms before the response was used for the recording interval. This was done to avoid brain activity artifacts related to the button press. Instances where a participant made no response before the presentation of the next stimulus were discarded.

For “participant response” scenarios (i.e., when the participant chose “yes” (y) or “no” (n) in response to the question: “Are the emotions the same?”)—brain activity from the first 2 s following the press of a button was recorded for each trial. In the event a participant responded within <2 s before the presentation of the next stimulus, that interval was used for the recoding time. Instances where a participant made no response before the presentation of the next stimulus were discarded.

A summary of fMRI contrasts is given in Table 2. A total of five subject-level contrast maps were created for all subjects using threshold free cluster enhancement (TFCE) of *z* > 2.3 and a cluster corrected significance threshold of *p* < 0.05. These modeled time series were convolved with the double gamma hemodynamic response function (dg-HRF), which was modeled from a combination of Gaussian functions. Group-level contrast maps were created using FMRIB’s Local Analysis of Mixed Effects (FLAME1). The thresholds for group level activation maps were created using TFCE of *z* > 1.5 and a cluster-corrected significance threshold of *p* < 0.05. The exact regions of brain activity were determined using the KNE96 coordinate space and Harvard-Oxford cortical and subcortical structural atlases.

**Table 2:** **Summary of functional magnetic resonance imaging (fMRI) contrasts**.

Task type	Option 1	Option 2	Option 3
EVDT-presentation of stimuli:	Two happy faces	Two neutral faces	One of each
EVDT-choice of the participant:	Yes	No	–

*A total of five subject-level contrast maps were created for all subjects. EVDT, emotional valence determination test*.

##### DTI

Voxel-based group differences were calculated for the *FA* images using Tract-Based Spatial Statistics (TBSS; Smith et al., 2006; Smith and Nichols, 2009; Cheon et al., 2011). TBSS linearly registered individual *FA* images in native space and then to the *FA* template via the FLIRT command of FSL. The resultant warping transformations were then used to convert images of diffusion (i.e., *FA*) to Montreal Neuroimaging Institute (MNI152) space with a spatial resolution of 1 × 1 × 1 mm. For statistical inference, including correction for multiple comparisons, permutation testing was used (Nichols and Holmes, 2002; Cheon et al., 2011) as implemented by RANDOMISE of the FSL software package. Five hundred permutations were performed for significant group differences at a threshold of 0.2; corresponding to *p* < 0.05, corrected for multiple comparisons using TFCE (Smith and Nichols, 2009; Cheon et al., 2011). WM tracts were identified using the Johns-Hopkins University ICBM-DTI-81 WM labels and probabilities of tract accuracy were assessed using the Johns-Hopkins University WM Tractography atlas.

### Behavioral Methodology and Analyses

All statistical calculations for behavioral analyses were performed using Rstudio Desktop (version 0.99.896, Rstudio, Inc., Boston, MA, USA).

#### Comparison of Test Performance Amongst Study Groups

Mann-Whitney U-tests with false discovery rate corrections of *α* = 0.05 for multiple comparisons to reach significance at *p* < 0.05 were used to evaluate differences in test performance between the study groups. A one-sample *t*-test with mu = 30 and two-sample U-test were used to calculate the group difference in MMSE score given that the variance for the NT group was zero. Both tests revealed a significant result. Additionally, the zero variance for the NT group on the MMSE is within reason for cognitively normal elderly individuals, as persons without cognitive deficits may be expected to receive a perfect score (30/30) as was seen here (Folstein et al., 1975).

#### Correlation of CFTs to Cognitive Test Performance

Pearson correlation coefficients, with false discovery rate corrections of *α* = 0.05 for multiple comparisons to reach significance at *p* < 0.05, were used to correlate CFT performance to performance on their respective cognitive tests.

#### Assessing Tests as Classifiers

Fischer Exact test, with corrections for multiple comparisons of *α* = 0.05 to reach significance at *p* < 0.05, were used to determine strength of each test as a classifier between CDDAT and NT.

## Results

### Behavioral Data

**Figure 2:**
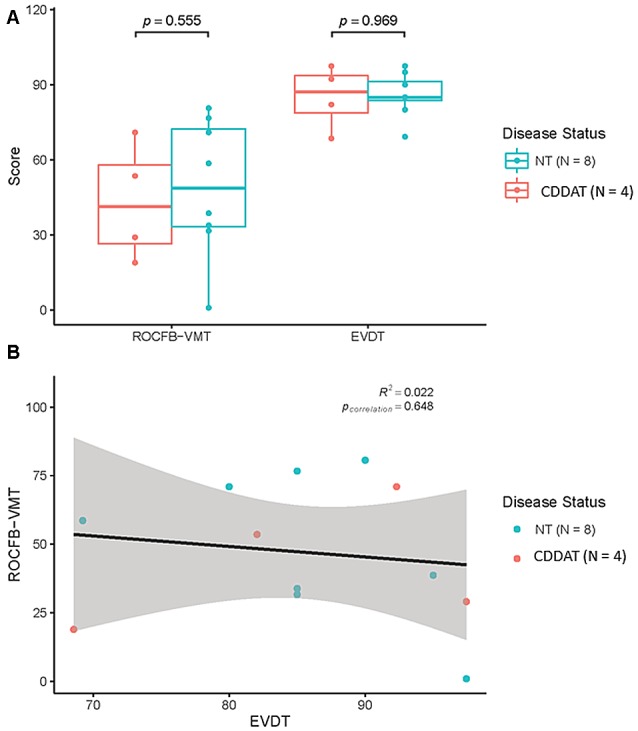
**(A)** Results of Rey-Osterrieth Complex Figure B-Visuospatial Memory Test (ROCFB-VMT) and EVDT. **(B)** ROCFB-VMT and EVDT performance were not correlated Pearson Correlation *r* = 0.022, *p*_correlation_ = 0.648. NT, Neurotypicals; CDDAT, Clinically diagnosed dementia of the Alzheimer Type.

We found no significant difference in median ROCFB-VMT or EVDT score for the two groups (NT = 58 ± 26.2 vs. CDDAT = 41 ± 23.6; Cohen’s *d* = −0.196, r-equivalent effect size = −0.0975, *p* = 0.555, NT = 85 ± 8.8 vs. CDDAT = 87 ± 13; Cohen’s *d* = 0.671, r-equivalent effect size = 0.318, *p* = 0.969, respectively). There was also no significant different RT for the EVDT (CDDAT = 2166 ± 461 ms vs. NT = 2084 ± 493 ms, not shown). Therefore, neither ROCFB-VMT nor EVDT function as a potential classifier between NT and CDDAT given their nearly identical overlap. All of these findings suggest that no observable difference in either emotional valence determination or visuospatial ability exists between these two groups at this stage of the disease. Additionally, we found no correlation between the ROCFB-VMT and EVDT performance (*R*^2^ = 0.022, *p* = 0.648). Thus, at this stage of the disease there appeared to be no connection between emotional valence determination ability and visuospatial memory decline; this finding, however, may be the result of a ceiling effect in participant performance. Behavioral results are summarized in Figure 2.

### Imaging Results

#### fMRI Results

There were no significant differences in areas of activation between groups upon stimulus presentation regardless of the emotional pairing displayed (data not shown). Furthermore, no differences in areas of activation were observed between groups with relation to when they chose “yes” or “no” in response to the question: “Are the emotions the same?”, thus indicating no fundamental difference in perception or processing of the faces (data not shown).

#### DTI Results

**Figure 3:**
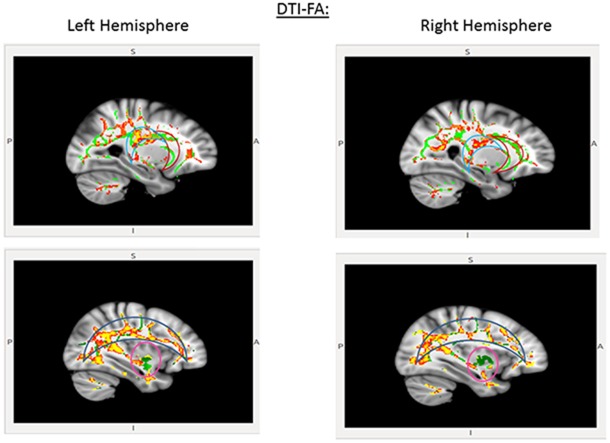
**Pertinent diffusion tensor imaging (DTI) results for the EVDT.** Significant differences were seen in the posterior cingulate cortex (PCC; light blue crescent, top) and superior longitudinal fasciculi (blue crescent, bottom), but not anterior cingulate (red crescent, top) or amygdaloidal connections (pink circle, bottom). Areas where NT > CDDAT for fractional anisotropy (FA) measures are shown in red. Green lines represent the FA skeleton. NT, Neurotypicals; CDDAT, Clinically Diagnosed Dementia of the Alzheimer Type.

CDDAT did not show higher *FA* values in any areas. NTs showed higher *FA* values than CDDATS in the right inferior longitudinal fasciculus (r-ILF), right posterior thalamic radiations (r-PTR) and the bilateral PCC (b-PCC) and superior longitudinal fasciculi (b-SLF). For the SLF, the differences between NTs and CDDATs were greater in the left than the right hemisphere, suggesting WM deterioration has occurred in the CDDAT group. No significant differences were observed in any of the three major amygdala connection pathways: the amygdalofugal, the stria terminalis, or the anterior commissure. Imaging results are summarized in Figure 3.

## Discussion

### Significance of Behavioral Findings

Consistent with our hypothesis and the previous findings of Albert et al. (1991) and Luzzi et al. (2007), CDDAT participants displayed no difference in accuracy or RT for either the ROCFB-VMT or the EVDT. These findings support the notion that non-verbal abilities, as measured by the ROCFB-VMT, and emotional valence determination, as measured by the EVDT, are largely intact in early-stage AD patients.

All of the subjects were combined together when analyzing the correlation of CFTs to cognitive test performance. Although it would be possible to add a categorical variable representing disease/no disease to the model to generate a multivariate linear model that controls for disease status, that would be ideal if the goal was to produce the most predictive model or if we were trying to demonstrate the causal dependence of the dependent variable on the independent variable.

However, there are several reasons we chose not to control for disease status. First, in principle, people who might apply these tests would likely not know disease status prior to administration. Thus, a model that controls for a variable that they don’t have available might confuse its application. Second, we are not proposing that CFT is causing cognitive test performance so much as they are simply correlated; that is, our goal is not necessarily to construct the most explanatory possible model. Controlling for disease status makes that impossible.

### Significance of fMRI Findings

Further support for our hypothesis comes from the fMRI results. We hypothesized that, in the absence of differences in behavioral performance, there would likely be no difference in brain activity. We also hypothesized that, if such a difference were seen, it would occur within the parietal lobes, amygdalae, or r-ACC based on fMRI findings in NTs reported by Fusar-Poli et al. (2009). The lack of differences between the brain activity patterns of the two groups upon stimulus presentation and during the selection of a response suggests that early-stage AD patients perceive the stimuli and choose a response in a fundamentally similar way to NTs, thereby strengthening the notion that these processes are not yet demonstrably affected by the disease.

### Significance of DTI Findings

Although our behavioral and functional imaging results seem promising with regard to the retention of non-verbal skill sets, a more concerning observation was made through DTI. Consistent with our hypothesis, we saw lower *FA* values in the b-SLF, especially within the left hemisphere, and b-PCC, but no differences in the amygdalae or r-ACC. While there were no significant differences in the major amygdala or anterior cingulate connection pathways, the changes in the SLF and the PCC are consistent with both the pathological progression of WM deterioration in AD (Braak and Braak, 1997; Bartzokis, 2004; Leech and Sharp, 2014) and proposed mechanisms of emotional valance (Maddock et al., 2003; Fusar-Poli et al., 2009).

In congruence with Indersmitten and Gur (2003), we propose the greater deterioration of the left-SLF tracts observed herein suggests a decline in processing efficiency for emotion, but a retention of the dominant circuit, which is served by tracts of the right-SLF. Finally, Kosaka et al. (2003) determined that the PCC plays an important role in face-processing for the transition of a face from being unrecognized to being acknowledged as familiar in NTs. Therefore, the early deterioration of PCC connecting fibers may play a significant role in AD patients’ impaired ability to recognize loved ones independent of semantic memory loss. Taken together, our findings further support the reverse demyelination hypothesis, proposed by Medina and Gaviria (2008), and Bartzokis’s (2004) assertion of the role of myelin damage predating neuronal loss and its possible role in exacerbating the progression of AD.

### Limitations

We acknowledge that the instances in which our behavioral analyses did not find a significant difference between our NT and CDDAT groups does not necessarily mean that impairments are not present at this time. Instead, it indicates that such deficits, if they exist, are less severe than those of semantic verbal fluency, which currently represents the most consistent and pronounced clinical manifestation of this disease at this stage (Henry et al., 2004). Additionally, the perfect score with zero variance obtained on the MMSE by our control group makes them an idealized group for comparison, but it also makes them less representative of the NT geriatric population as a whole. It is important to note, however, that they were not intentionally selected to obtain a perfect score and that this is an incidental result.

As previously mentioned, the inability to directly correlate ROCFB-VMT: EVDT performance may be due to a ceiling effect. Replicating this experiment with a more difficult visuospatial test, such as the Rey Osterrieth Complex figure A (ROCFA) may result in an observable difference between NTs and early-stage CDDAT, but given that a similar correlation has been established for the MLT and ROCFB in moderate-stage AD by Luzzi et al. (2007) such an observation may be more academic than clinically relevant.

It is important to note, however, that since the etiology of AD is unknown and requires post mortem confirmation it is possible that our findings may not extend to all individuals affected with the ailment. Other potential cofounders could be differences in the demographics and comorbidities in the two groups. Due to restrictions related to the approved Human Protections Board protocol, we do not have any additional medical or demographic information (e.g., marital status, list of medications or medical comorbidities that are not of a neurologic or psychiatric nature) on our participants than what is currently described. An alternative interpretation is that although there is significant structural damage, the lack of functional differences between the groups may represent a type of structural compensation independent of reverse demyelination. Conversely, these observations may be rooted in fundamental issues of neurodegeneration and may therefore extend to other neurodegenerative diseases making these findings simultaneously of larger interest to the field as a whole, but less exclusive to AD. Additional investigations will be necessary to access the reproducibility of these findings in other populations as well as their specificity to AD.

## Conclusion

While we observed that both groups performed similarly on both skill sets, we found no correlation between the two, due in part to the massive variations in ROCFB-VMT scores. Yet, since both groups scored within normative ranges after adjusting for age and education (Becker et al., 1987), we conclude that both skill sets are intact within these participants at this stage of the disease. Furthermore, there were no significant differences in BOLD fMRI activation with regard to either stimulus presentation or participant response, strengthening our assertion that these skill sets are, as of yet, unaffected. Lastly, these findings are consistent with the previous work by Luzzi et al. (2007). The deterioration of WM tracts within the b-SLF and b-PCC may foreshadow the impending decline of these functions, as would be consistent with clinical observations (Moore and Wyke, 1984; Haupt et al., 1991; Liu et al., 1991) and autopsy findings (Braak and Braak, 1997) for the progression of the disease.

In conclusion, our discoveries highlight the potential of WM tractography as a presymptomatic biomarker for AD. Based on our observations we suggest that when caring for patients with suspected early-stage AD, use direct/pronounced body language and facial expressions over verbal commands whenever possible. Emphasizing non-verbally mediated social cues has the added benefit of strengthening social interaction, which may help slow the progression of symptoms (Bennett et al., 2006) and reduce the risk of depression (Yaffe et al., 1999).

## Author Contributions

RR created and executed the project, analyzed and interpreted the data, and wrote the manuscript. RCA and DF assisted in the neuroimaging experimental design and analyses. AGM and HMRK assisted in experimental design and statistical analyses and interpretation. PL, PJ, GH, KL, JC and JT determined participant eligibility and consulted on medical interpretation and significance. PHR and MB provided consultation on experimental design and interpretation.

## Conflict of Interest Statement

The authors declare that the research was conducted in the absence of any commercial or financial relationships that could be construed as a potential conflict of interest.

## Footnotes

^1^www.fmrib.ox.ac.uk/fsl
